# Optimal Transport Scenario With Rotary Air Transport for Access to Endovascular Therapy Considering Patient Outcomes and Cost: A Modeling Study

**DOI:** 10.3389/fneur.2021.768381

**Published:** 2021-12-17

**Authors:** Ashlee Wheaton, Patrick T. Fok, Jessalyn K. Holodinsky, Peter Vanberkel, David Volders, Noreen Kamal

**Affiliations:** ^1^Department of Industrial Engineering, Dalhousie University, Halifax, NS, Canada; ^2^Division of Emergency Medical Services (EMS), Department of Emergency Medicine, Dalhousie University, Halifax, NS, Canada; ^3^Department of Clinical Neurosciences, Cummings School of Medicine, University of Calgary, Calgary, AB, Canada; ^4^Department of Diagnostic Radiology, Faculty of Medicine, Dalhousie University, Halifax, NS, Canada

**Keywords:** ischemic stroke, endovascular treatment, patient outcome, transport cost, air transport, optimization, modeling

## Abstract

**Background and Purpose:** For an ischemic stroke patient whose onset occurs outside of the catchment area of a hospital that is capable of Endovascular Treatment (EVT) and whose stroke is suspected to be caused by a large vessel occlusion (LVO), a transportation dilemma exists. Bypassing the nearest stroke hospital will delay Alteplase but expedite EVT. Not bypassing allows for confirmation of an LVO diagnosis before transfer to an EVT-enabled facility, but ultimately delays EVT. Air transport can reduce a patient's overall time to treatment however, it is costly. We expanded on an existing model to predict where Drip-and-Ship vs. Mothership provides better outcomes by including rotary air transport, and we also included prediction of where either the transport method was most cost effective.

**Methods:** An existing model predicts the outcome of patients who screen positive for an LVO in the field based on how they were transported, Drip-and-Ship (alteplase-only facility first, then EVT-enabled facility) or Mothership (direct to EVT-enabled facility). In our model, the addition of rotary wing transportation was conditionally applied to inter-facility transfer scenarios where it provided a time advantage. Both patient outcome and transport cost functions were developed for Mothership and Drip-and-Ship strategies including transfers via either ground or air depending on the conditional probabilities. Experiments to model real world scenarios are presented by varying the driving time between the alteplase-only and EVT-enabled facility, time to treatment efficiencies at the alteplase-only facility, and EVT eligibility for LVO patients. Patient outcome and transport costs were evaluated for Mothership and Drip-and-Ship strategies.

**Results:** The results are presented in temporospatial diagrams that are color coded to indicate which strategy optimizes the objectives. In most regions, there was overall agreement between the optimal solution when considering patient outcomes or transport costs. Small regions exist where outcome and cost are divergent; however, the difference between the divergence in Mothership and Drip-and-Ship in these regions is marginal.

**Conclusions:** The optimal transport method can be optimized for both patient outcomes and transport costs.

## Introduction

The time between stroke onset and treatment remains the largest indicator of outcomes for ischemic stroke patients (1, 2). Both alteplase (3, 4) and endovascular thrombectomy (5–9) (EVT) are proven effective treatments for ischemic strokes caused by a large vessel occlusion (LVO); however, EVT treatment results in better outcomes than alteplase alone for these patients. Not all hospitals can offer EVT as the procedure requires specialized personnel and equipment. Studies have been done to assess which strategy is best for transporting a patient whose stroke onset occurs outside the direct catchment area of an EVT-enabled facility (10–16). Two strategies exist for transporting these patients, the Drip-and-Ship strategy (alteplase-only facility first, then transfer to an EVT-enabled facility), and the Mothership strategy (direct to an EVT-enabled facility). However, these studies have rarely included the use of air transportation between facilities. In practice, aeromedical transport is often employed to transfer acute stroke patients between facilities to access EVT when there are long distances between the facilities, and a ground ambulance is used to transport patients from the scene. Inter-facility transfer via aeromedical transport allows for quicker access to an EVT-enabled facility in the Drip-and-Ship strategy but is more costly than ground transport.

The Drip-and-Ship transport strategy also allows for greater specificity for confirmed ischemic patients with an LVO transported to the EVT-enabled facility. This increase in specificity is because patients will have Computed Tomography (CT) imaging and a CT Angiogram (CTA) to confirm an LVO. The mothership strategy would transport all patients who screen positive using a pre-hospital screening tool, including ischemic stroke patients with non-LVO, hemorrhagic stroke patients, and stroke mimics. Therefore, the Drip-and-Ship strategy can potentially provide transport cost savings in some cases, which may offset the high cost of an air ambulance for inter-facility transport. To test this hypothesis, we expanded on an existing model to assess the optimal transport strategy, Drip-and-Ship or Mothership, based on the seemingly opposing objectives of both transport costs and patient outcomes. We built upon an existing model to predict which transport method provides better patient outcomes by including rotary air transport, and we also included prediction of where either the transport method was most cost effective.

## Methods

A previously published conditional probability model (11), included the formulation of probabilities of an excellent outcome for a suspected LVO ischemic stroke patient based on which strategy was used to transport the patient to treatment, Drip-and-Ship or Mothership. We have expanded this model to include the use of air ambulances by developing a probability of inter-facility transfer via rotary air and conditionally applying it to scenarios where air transport provides a time advantage. We have also accounted for contraindications to EVT treatment in this study, as not all LVO patients will receive EVT, and this impacts both patient outcomes and transport costs. Alongside the evaluation of patient outcomes, we developed transport cost formulations for both the Drip-and-Ship and Mothership strategies, and both objectives are presented and compared.

### Probability of Inter-facility Transfer via Air Ambulance

The use of fixed-wing air transport differs from a rotary-wing air transport logistically; specifically, there are differences in lead times, landing capabilities, and cruising speeds. Generally, rotary-wing transport is used most extensively for rapid transfer of acute stroke patients. Therefore, we have modeled air transportation by assuming the speed and logistics of a rotary-wing air ambulance only.

There are external factors which impact the ability to transport a patient via air: airworthy weather and resource availability. The probability of air resource availability is meant to model scenarios when air transport is requested but declined because the resource is currently unavailable; for example, the aircraft is currently on another mission, or the crew is unavailable.

Air transport may not always be initiated for a transfer if the time advantage it provides is minimal. We modeled the likelihood of considering the use of air transport given the time advantage using a piecewise linear function. Where the likelihood of considering an air transport for an inter-facility transfer is zero until the time advantage it provides is greater than an assumed threshold. Until the time advantage surpasses a second assumed threshold the probability of considering air transport for an inter-facility transfer is calculated using linear interpolation between the two thresholds and the time advantage for a given scenario. Beyond the second assumed threshold the probability of considering air transport for an inter-facility transfer is one. This formulation means that the probability of considering air transport varies based on the distance between the alteplase-enabled and EVT-enabled facilities. The time advantage is the time to the endovascular center via air (includes the alarm to wheels up time, the time on the ground at the thrombolysis center, the air travel time to the thrombolysis center, and the travel time to the endovascular center) minus the ground transport travel time between the centers. The minimum time advantage to begin considering air transport is 10 min (the first threshold) and the maximum time advantage when air transport is always considered is 40 min (the second threshold). The formulation for the piecewise linear function is provided in Table 1.

**Table 1:** Formulation for probability of using air transport in the Drip-and-Ship Strategy.

**Probability**	**Formulation**	**Rationale**
Pr {Air}	Pr{Air Consideration} · Pr{Airworthy Weather} · Pr{Air Resource Avialibility}	The product of the three factors which impact the likelihood of an inter-facility transfer occurring via air make up the overall probability of an inter-facility transfer via air.
Pr {Ground}	1 − Pr{*Air*}	The probability of an inter-facility transfer occurring via ground is the complement of it occurring via air. The underlying assumption we made here is that if a transfer cannot occur via air it will occur via ground.
Pr {Air Consideration | t_tPA only to EVT Enabled_}	$\{\begin{array}{ccc} 0 & , & if\;\Delta T\leq \Delta T_{1}\\ \frac{\Delta T-\Delta T_{1}}{\Delta T_{2}-\Delta T_{1}} & , & if\;\Delta T_{1}<\Delta T<\Delta T_{2}\\ 1 & , & if\;\Delta T\geq \Delta T_{2} \end{array}$	The probability of air consideration for an inter-facility transfer is dependent on the time advantage air transportation can offer a scenario. We have assigned this time advantage to the *T* variable. The probability of air consideration is formulated as a piecewise function. Up to the initial threshold of *T*_1_, which in the model is assigned 10 min, the time advantage provided by air is considered negligible. At and below this point the probability of considering air for the inter-facility transfer is 0. Beyond a second threshold of *T*_2_, which in the model is assigned 40 min, the time advantage is considered undeniable. At and beyond this point the probability of considering air for the inter-facility transfer is 1. Between these two thresholds, a linear interpolation between the two thresholds is used to determine the probability of air consideration.
Pr {Airworthy Weather}	0.904	The probability of airworthy weather for an inter-facility transfer via air is assumed to be constant. This value comes from a study done on aborted air ambulance missions in Nova Scotia, Canada (17).
Pr {Air Resource Availability}	0.965	The probability of air resource availability for an inter-facility transfer via air is assumed to be constant. This value comes from a study done on aborted air ambulance missions in Nova Scotia, Canada (17).

*PSC is the Primary Stroke Center that is defined as a facility only capable of alteplase treatment; CSC is the Comprehensive Stroke Center that is defined as a EVT enabled facility (EVT, Endovascular Thrombectomy)*.

The probability of a transfer occurring via air transport was modeled as the product of the probabilities of airworthy weather, air resource availability, and consideration of air transport. The probability of a transfer occurring via ground is the complement of the probability that the transfer occurs via air. The underlying assumption of this method infers that if an inter-facility transfer cannot occur via air, it will occur via ground. Table 1 shows the formulation and rationale for the probability of transferring via air in the Drip-and-Ship strategy. The definition of the variables found in this formulation are shown in **Table 3**.

### Patient Outcome Modeling

Patient outcome modeling was formulated using a previously published study (11), where the probability of an excellent outcome given that a patient has screened positive in the field for an LVO ischemic stroke was derived using data from randomized clinical trials for both alteplase and EVT. In this formulation an excellent outcome is defined as a 0–1 score on the modified Rankin Scale at 90 days. To model the uncertainty of a pre-hospital screening tool diagnosis of a suspected LVO ischemic stroke, this study accounted for other possible final diagnoses. The other possible diagnoses for those that screen positive using a pre-hospital screening tool are: ischemic stroke with a non-LVO, intracerebral hemorrhage, and a stroke mimics. The distribution of these final diagnoses were consistent with a previous study (11) to ensure consistency of results.

We have adapted the previous formulation (11) for our model to calculate the probability of an excellent outcome assuming a Drip-and-Ship strategy that conditionally includes the use of inter-facility transfer via air transport. For the proportion of patients that are transferred between the alteplase-only facility and the EVT-enabled facility via ground (probability of ground transfer), the formulation remains the same. For the proportion of patients that are transferred via air, the time from symptom onset to groin puncture uses the transport time for air transport. The sum product of the respective probability of an inter-facility transfer via air and ground transport represents the expected probability of an excellent outcome for the Drip-and-Ship strategy.

### Transport Cost Modeling

Transport cost functions were formulated for both the Mothership and Drip-and-Ship strategies using fixed and variable cost elements. Fixed costs were assumed to account for vehicle insurance, depreciation of vehicle value, salary of ambulance dispatchers, and other overhead costs associated with delivering ambulance services. Variable costs were assumed to account for fuel, paramedic salary, vehicle maintenance and other distance dependent costs.

Since the Mothership strategy can only be accomplished via ground transportation, the formulation of the expected transport cost is a straightforward linear equation. The Mothership formulation equates to the assumed fixed cost of transportation plus the variable cost element given the distance between the scene and the EVT-enabled facility.

As the Drip-and-Ship Strategy includes a stop at the closest alteplase-only facility to allow for diagnosis, patients who do not receive an LVO diagnosis or are ineligible for EVT do not require further transport to an EVT-enabled facility. The opportunity to rule out the need for further transport of some patients equates to cost savings in the Drip-and-Ship strategy. Since the inter-facility transport can occur via air or via ground transport, the expected cost of inter-facility transport is calculated using the probabilities of air and ground transport. This is multiplied by the proportion of LVO patients who are eligible for EVT and added to the cost of the ground transport between the scene and the alteplase-only facility to model the expected cost of the Drip-and-Ship strategy.

Displacing a ground ambulance over large distances impacts the EMS system in more than just a monetary nature. We have doubled the variable cost of ground ambulances to model the monetary impact as well as a serve quantifiable measure of the strain that displacing a ground ambulance has on an EMS system. The variable portion of air transport was not doubled as the displacement of an air resources over large distances is the purpose of an air transport system. Table 2 shows the formulation for determining transport cost using both the Drip-and-Ship and Mothership transport scenario. The definitions for the variables found in this formulation are listed in Table 3.

**Table 2:** Formulation for determining transport cost using both the Drip-and-Ship and Mothership transport scenario.

**Cost**	**Formulation**	**Rationale**
TC {Mothership}	*F*_*G*_ + (2 · *V*_*G*_ · *D*_*scene to CSC*_)	The Mothership transport cost is completed using ground ambulances only since air ambulances are assumed to never land on-scene.
TC {Drip and Ship}	TC{*D*_*scene to PSC*_} + α · *Y* · [(Pr{*Ground*} · *TC* {*D*_*PSC to CSC*_}|*Ground*)+ (Pr{*Air*} · *TC* {*D*_*PSC to CSC*_}|*Air*)]	The Drip and Ship transport cost consists of two legs of transport. The first from the scene to PSC for all suspected LVO patients. The inter-facility transfer is only required for confirmed LVO patients who are eligible for EVT and can occur via ground or air transportation. The α and Y variables represent the proportion of LVO patients and EVT eligible LVO patients, respectively.
TC {*D*_*scene to PSC*_}	*F*_*G*_ + (2 · *V*_*G*_ · *D*_*scene to PSC*_)	This represents the scene to tPA-only facility transport in the Drip and Ship strategy which is completed using ground ambulance only since air ambulances are assumed to never land on-scene.
*TC* {*D*_*PSC to CSC*_}|*Ground*	$F_{G}^{{}^{\prime }}+\left(2\cdot V_{G}\cdot D_{PSC\;to\;CSC}\right)$	This represents the inter-facility transfer transport cost if it occurs via a ground ambulance. A prime version of the fixed ground cost is applied in this case to model the idea that if ground transport is used, initiating it between hospital facilities likely costs the system less than scene to hospital transport.
*TC* {*D*_*PSC to CSC*_}|*Air*	*F*_*A*_ + (*V*_*A*_ · (*D*_*airbase to PSC*_ + *D*_*PSC to CSC*_))	This represents the inter-facility transfer transport cost if it occurs via an air ambulance. The variable cost of air transport accounts for transport from the airbase to the tPA-only facility and the transport between facilities.

*PSC is the Primary Stroke Center that is defined as a facility only capable of alteplase treatment; CSC is the Comprehensive Stroke Center that is defined as a EVT enabled facility (EVT, Endovascular Thrombectomy)*.

**Table 3:** Definition for variables used in formulations.

**Variable**	**Definition**
Pr {Air}:	Probability of transport via air
Pr{*Air Consideration*}	Probability that air transport provides time advantage
Pr{*Airworthy Weather*}	Probability of air transport weather conditions
Pr{*Air Resource Avialibility*}	Probability of air resource availability
Pr {Ground}	Probability of ground transport
Δ*T* = *t*_*PSC to CSC*|*G*_ − (*t*_*alarm to wheels up*_ + *t*_*airbase to PSC*_ + *t*_*on ground @ PSC*_ + *t*_*PSC to CSC*|*A*_)	Time advantage of used air transport
*t*_*PSC to CSC*|*G*_	Travel time from PSC to CSC via ground transport
*t*_*alarm to wheels up*_	Time from receiving the alarm that the patient will be transported by air to the time that the helicopter is in the air
*t*_*airbase to PSC*_	Air travel time from airbase to PSC
*t*_*on ground @ PSC*_	Time that the helicopter is at the PSC
*t*_*on ground @ PSC*_	Air travel time from PSC to CSC
Δ*T*_1_	Minimum time advantage that is required for when air transport is considered
Δ*T*_2_	Time advantage when air transported is always considered
Pr {Airworthy Weather}	Probability that the weather will permit air transport
Pr {Air Resource Availability}	Probability of air resource availability
TC {Mothership}	Transport cost via mothership
*F*_*G*_	Fixed cost for ground transport
*V*_*G*_	Variable cost per km for ground transport
*D*_*scene to EVT enabled*_	Distance from scene to CSC (km)
TC {Drip and Ship}	Transport cost via drip-and-ship
*TC*{*D*_*scene to tPA only*_}	Transport cost from scene to PSC
α	Proportion of LVO patients picked by stroke screening tool
*Y*	Proportion of LVO patients eligible for EVT
TC {*D*_*PSC to CSC*_}|*Ground*	Transport cost from PSC to CSC via ground
TC {*D*_*PSC to CSC*_}|*Air*	Transport cost from PSC to CSC via air
TC {*D*_*scene to PSC*_}	Transport cost from scene to PSC
$F_{G}^{{}^{\prime }}$	Fixed ground transport cost for transfers
*D*_*PSC to CSC*_	Distance from PSC to CSC (km)
*F*_*A*_	Fixed cost for air transport
*V*_*A*_	Variable air transport cost per km
*D*_*airbase to PSC*_	Distance between the airbase and CSC km

*PSC is the Primary Stroke Center that is defined as a facility only capable of alteplase treatment; CSC is the Comprehensive Stroke Centre that is defined as a EVT enabled facility (EVT, Endovascular Thrombectomy)*.

All transport costs that were used for this study are provided in Table 4. The ground transport costs were estimated using the amount that three Canadian Provinces (Nova Scotia, British Columbia, and New Brunswick) charge non-residents for ambulance services. The cost of air transport was estimated using the amount that British Columbia and Alberta charge non-residents for rotary wing ambulance services. These rates were posted to their websites at the beginning of 2021.

**Table 4:** Assumed parameters used in the model for the generated results.

**Input parameter**	**Value**
Onset to first medical contact	30 min
Response time	15 min
On scene time	15 min
Door-to-needle time	Efficient PSC scenarios: 30 min Inefficient PSC scenarios: 60 min
Needle-to-door-out time	Efficient PSC Scenarios: 20 min Inefficient PSC Scenarios: 60 min
Air ambulance alarm to wheels-up	15 min
Air ambulance on ground at PSC	20 min
Probability of airworthy weather	90.4%
Probability of air resource availability	96.5%
Speed of ground ambulance	80 km/hr
Speed of air ambulance	254 km/hr
Fixed cost of ground transport from scene	500.00 CAD
Fixed cost of inter-facility transfer via ground	400.00 CAD
Fixed cost of air transport	2,500.00 CAD
Variable cost of ground transport	5.00 CAD/km
Variable cost of air transport	12.00 CAD/km

*PSC is the Primary Stroke Center that is defined as a facility only capable of alteplase treatment; CSC is the Comprehensive Stroke Center that is defined as an EVT enabled facility (EVT, Endovascular Thrombectomy)*.

### Visualizations

The results are presented using temporospatial diagrams that are consistent with previous studies (11, 16, 18). These allow for a generalized solution which can be used to indicate the results for different geographic regions.

The catchment area of the alteplase-only facility is illustrated by a series of concentric circles at the center of which is a circle representing the location of the facility. The diamond at the bottom of the diagram represents the location of the EVT-enabled facility. Yellow and white arcs appear in some of the diagrams presented. Patients beyond yellow arc will have an onset-to-needle time beyond 4.5-h at the EVT-enabled facility and patients beyond the white arc will have an onset-to-needle time beyond 4.5-h at the alteplase-only facility. These regions model remote rural regions which are likely very rare in most geographies.

We developed these visualizations using MATLAB software (version R2020a, Mathworks Inc., Natick, MA). The 2D plotting function was applied to output the temporospatial diagrams. Each pixel in the output diagrams represents a potential scene location within the catchment area of the alteplase-only facility. The distance and time relative to the alteplase-only and EVT-enabled facility are calculated for each scene location and passed to sub-routines which calculate the probability of an excellent outcome and expected transport costs for a patient picked up at each scene. The temporospatial diagrams are color coded based on the output of these sub-routines.

There are four possible combinations of the two objectives (outcomes and cost) in this model. Each objective is optimized with either the Mothership or Drip-and-Ship strategy. If both objectives agree on the transport strategy the scene location is color coded either red for Drip-and-Ship, or green for Mothership. If the two objectives disagree on the transport strategy the scene location is said to be in a *divergent region* and is color coded either purple or blue. Purple indicates that Drip-and-Ship is associated with a higher probability of an excellent patient outcome, but Mothership is expected to be least expensive. Blue indicates that the opposite of this is true.

#### Scenarios and Constants

There were several scenarios that were run to illustrate the results of our model. These scenarios were created by varying times between the alteplase-only and EVT-enabled facilities, efficiencies at the alteplase-only facilities, and EVT eligibility for LVO patients. Two efficiency scenarios are modeled for the alteplase-only facility by varying the door-to-needle (DTN) and needle-to-door-out (NTDO) times. An inefficient alteplase-only facility is modeled with DTN and NTDO times of 60-min each. An efficient alteplase-only facility is modeled with a DTN time of 30 min and a NTDO time of 20 min. It should be noted that NTDO only applies to ground transport. For rotary air transport the time from thrombolysis treatment and leaving the PSC, we consider the alarm-to-wheels-up time and air-ambulance-on-ground-at-PSC that are constant at 15 min and 20 min, and these two values are added to the travel time from airbase to PSC. These efficient scenarios may not be achieved yet in some health systems, but they are the current benchmark in Canadian Best Practice Guidelines. LAMS was used as the prehospital LVO screening tool for all of our models, and a LAMS of 4 or more is considered positive. All of the constants used in our scenarios are provided in Table 4. The probability of airworthy weather for air transport and the probability of air resource availability were assumed to be 90.4 and 96.5%, respectively (19). It was assumed a LAMS of 4 or more will yield the following final diagnosis distribution: 45.38% ischemic stroke with an LVO, 10.92% ischemic stroke with a non-LVO, 34.45% intracerebral hemorrhage, and 9.24% stroke mimics (11).

The proportion of LVO ischemic stroke patients that received EVT was changed to create two scenarios. These scenarios used 50 and 70% of LVO ischemic stroke patients being eligible for EVT. The eligibility for alteplase was not changed, and our model assumed that all ischemic stroke patients, LVO and non-LVO, who are within 4.5 h of stroke onset received alteplase, which is consistent with the previous model (11).

To convert between ground ambulance drive times and air transport flying times we have assumed an average cruising speed for both modes of transport. An average cruising speed of 80 km/hr was assumed for ground ambulance transport. We used the average cruising speed of a Sikorsky S-76C+ model helicopter of 254 km/hr for the average air ambulance cruising speed. This helicopter model was chosen as it is employed to deliver aeromedical transport in Nova Scotia, Canada by EHS (Emergency Health Services) LifeFlight.

The probability of inter-facility transfer via air transport is calculated based on the ground travel time between the alteplase-only and EVT-enabled facilities. Four one-hour increments of travel time between these two facilities are shown for each scenario presented in the results. The probability of inter-facility transfer via air transport for the first two time increments (60 min and 120 min between facilities via ground) is 0% as air transport does not provide a time advantage of >10 min. The probabilities of inter-facility transfer via air transport for the next two time increments (180 min and 240 min between facilities via ground) are 62.79 and 87.15%, respectively.

## Results

**Figure 1:**
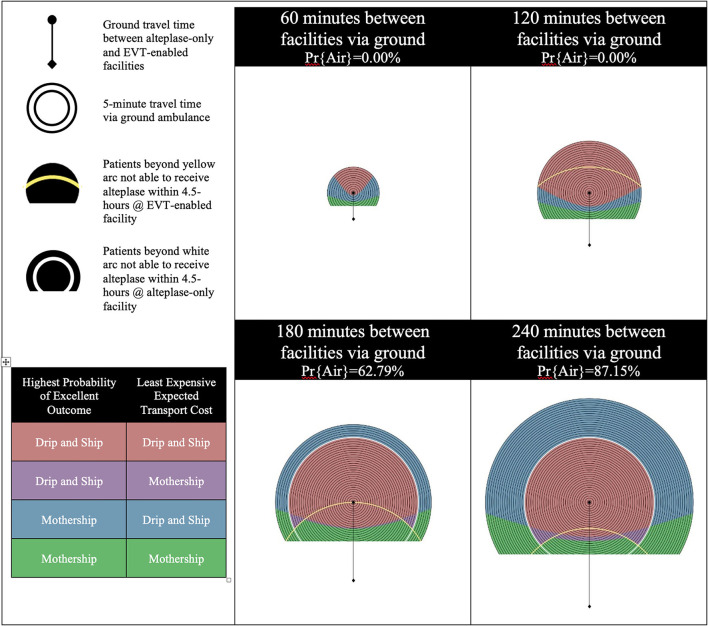
Scenario A—inefficient alteplase-only facility and 50% EVT eligibility for LVO patients. LVO, Large Vessel Occlusion; Pr{Air}, Probability of air transport.

In total there were four scenarios with varying alteplase-only facility efficiency and proportion of LVO patients treated with EVT. Each of these scenarios had four distances between the alteplase-only facility and EVT-enabled facility. Scenario A is our base case scenario, which models an inefficient alteplase-only facility and 50% EVT eligibility for confirmed LVO patients. Figure 1 shows the results of experiments run for Scenario A. These results illustrate a large region where Drip-and-Ship is preferred in both objectives, patient outcome and transport cost, color coded with red. However, regions of divergence show up between the two facilities in all four time increments shown for this scenario. This is primarily due to the backtracking involved with the Drip-and-Ship strategy in this region, as the patient needs to be transported away from the EVT-enabled facility to first be transported to the alteplase-only facility. In the 60 and 120 min increments this region of divergence is colored blue indicating that Mothership is associated with the highest probability of an excellent outcome, but Drip-and-Ship is expected to be least expensive. As the time between facilities increases to 180 and 240 min, this region of divergence changes to purple indicating that Drip-and-Ship is associated with the highest probability of an excellent outcome, but Mothership is expected to be least expensive. This shift is caused by the high likelihood of inter-facility transfer via air transport when the distance between facilities is longer as patients reaching EVT sooner results in better LVO patient outcomes but comes at a cost. A large portion near the bottom of all four diagrams in this scenario indicates that Mothership is preferred by both objectives, which is largely due to inefficiencies at the alteplase-only facility and proximity to the EVT-enabled facility. We also see a region of blue divergence in the 180 and 240 min between facilities beyond the white arc, which indicates patients in this region would have an symptom onset-to-needle time over the 4.5-h threshold for alteplase at the alteplase-only facility. This means LVO patients in this region are only eligible for EVT treatment, which explains why Mothership is preferred for patient outcome. However, this region is furthest away from the EVT-enabled facility and therefore Drip-and-Ship results in the least expensive transport cost, which is due to the opportunity to rule out the need to transfer a portion of patients (those without an LVO final diagnosis and for patients who possess contraindications to the EVT procedure).

**Figure 2:**
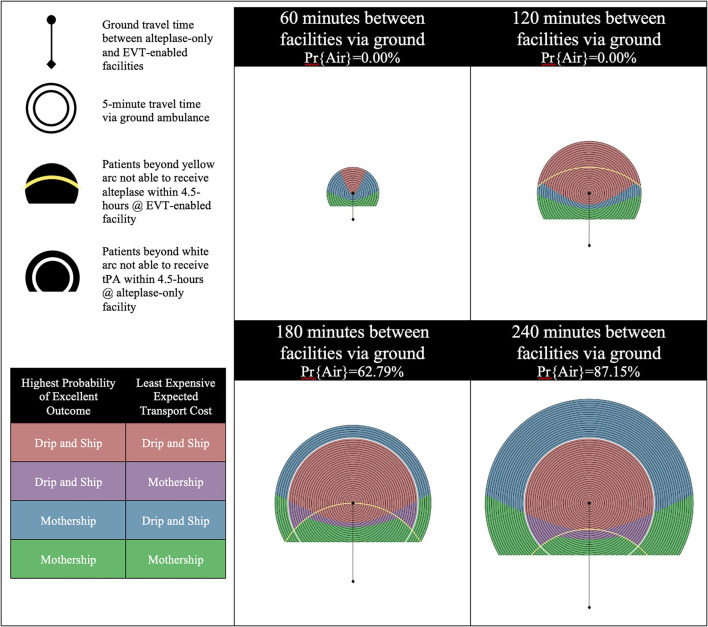
Scenario B—Inefficient alteplase-only facility and 70% EVT eligibility for LVO patients. LVO, Large Vessel Occlusion; Pr{Air}, Probability of air transport.

In Scenario B, the alteplase-only facility remains inefficient as the EVT eligibility for LVO ischemic stroke patients is increased from 50% in Scenario A to 70%. Figure 2 shows the results of experiments run for Scenario B. An increase in the proportion of LVO patients eligible for EVT results in two changes: (1) improved LVO patient outcomes and, (2) increased expected Drip-and-Ship transport cost as more patients require inter-facility transfer for EVT. This steepens the tradeoff between patient outcome and transport cost, and the impact of this differs for divergent regions based on the likelihood of inter-facility transfer via air transport. In the 60 and 120 min increments an increase in EVT eligibility causes a shift toward Mothership for both the patient outcome and transport cost objectives. This is illustrated between the results of Scenario A and B as a shift from red to blue color coding and blue to green color coding. In the 180 and 240 min increments an increase in EVT eligibility has little impact on patient outcomes but causes the expected cost of Drip and Ship to increase. This is illustrated between the results of Scenario A and B as a shift from red to purple color coding.

**Figure 3:**
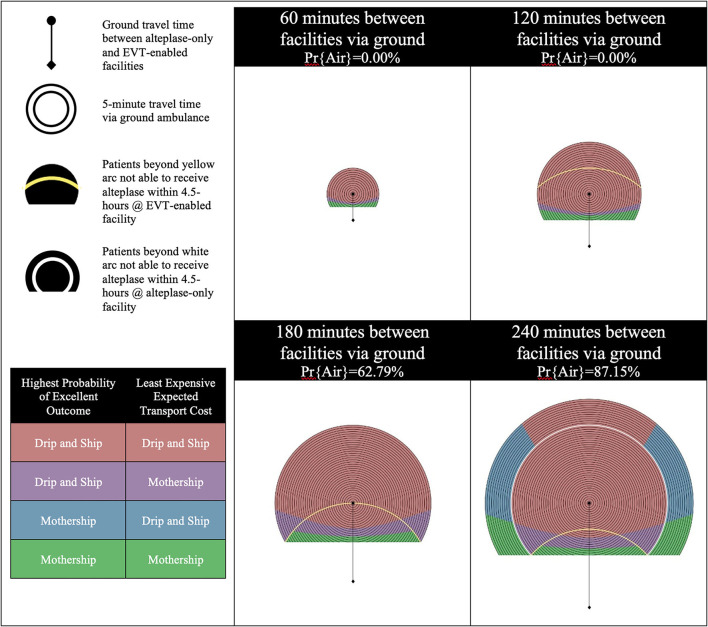
Scenario C—Efficient alteplase-only facility and 50% EVT eligibility for LVO patients. LVO, Large Vessel Occlusion; Pr{Air}, Probability of air transport.

Scenario C models an efficient alteplase-only facility and 50% EVT eligibility for confirmed LVO patients. Figure 3 shows the results of experiments run for Scenario C. Increased efficiency at the alteplase-only facility impacts only the patient outcome objective while the transport cost objective remains the same. Far less divergence is noted when efficiency is increased at the alteplase-only facility as all four increments are predominately colored, indicating that Drip-and-Ship is preferred in both objectives. This is illustrated by comparing the results of Scenario A and C as a shift from blue to red color coding. Increasing the efficiency at the alteplase-only facility reduces a patient's onset-to-needle and onset-to-puncture time which therefore positively impacts their outcome. An inter-facility transfer via air further reduces a patient's onset-to-puncture time, therefore in the 180 and 240 min increments where the likelihood of this is high, the Drip-and-Ship strategy produces favorable results in the majority region. This is even true in some regions outside the white arc in the 240-min increment, where the alteplase-only facility can only offer patients access to inter-facility transfer via air transport and no alteplase treatment since patients arrive after 4.5 h from onset.

**Figure 4:**
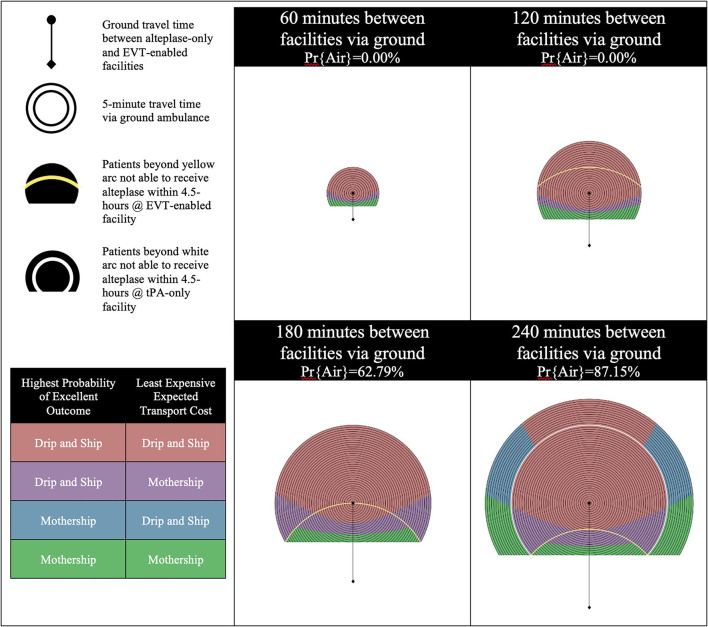
Scenario D—Efficient alteplase-only facility and 70% EVT eligibility for LVO patients. LVO, Large Vessel Occlusion; Pr{Air}, Probability of air transport.

In Scenario D, the alteplase-only facility remains efficient as the EVT eligibility for LVO patients is increased from 50 to 70%. Figure 4 shows the results of experiments run for Scenario D. An increase in EVT eligibility for LVO patients has less impact on patient outcomes when efficiency at the alteplase-only facility is increased. This is illustrated by the comparison of the differences between Scenarios A and B with the differences between Scenarios C and D. We still see a slight impact on the transport cost associated with more patients requiring inter-facility transfer for EVT but far less impact on patient outcomes can be observed.

An advanced analysis of these results was completed to provide insights from the model with a higher level of granularity. During this analysis, several points on the temporospatial diagrams were selected and the numerical results compared. Points from each color coding were used to analyze the magnitude of the difference between the Mothership and Drip-and-Ship strategies for both objectives. This analysis showed that in the divergent regions, the differences between the two strategies in these regions are marginal for at least one objective, which means that the divergence was less severe and one of the two strategies was preferred. In the purple areas of divergence, where Drip-and-Ship resulted in better patient outcomes, but Mothership resulted in more cost effectiveness. The difference in patient outcomes with Drip-and-Ship is more than 5% better probability of good outcomes; for example, one random point showed 35.04% probability of good outcomes with Drip-and-Ship vs. 29.06% probability of good outcomes with Mothership. However, the cost benefit with Mothership was < $300; for example, in the same random point, the cost with Drip-and-Ship was $3,537, but the cost with Mothership was $3,251. Conversely, in the blue areas of divergence, where Mothership resulted in better patient outcomes, but Drip-and-Ship results in better cost effectiveness, the difference in patient outcomes was <0.1%; for example, in a random location in the blue area, the probability of good outcomes with Drip-and-Ship was 28.65% compared to 28.67% with Mothership. However, the cost benefit with Drip-and-Ship was over $2,000; for example, in the same location discussed for patient outcomes, the cost of transport with Drip-and-Ship is $4,444 compared with $6,566 with Mothership.

## Discussion

The results of this study indicate that most scenarios can be optimized for both patient outcome and transport costs with the same transport strategy, Mothership or Drip-and-Ship. In regions where divergence exists, one or both objectives show a marginal difference between transport strategies. The marginal difference in regions of divergence indicates the difference between the strategies can likely be recovered as a result of the downstream hospital cost savings associated with better patient outcomes (17, 20–22).

The addition of inter-facility transfers via air within the Drip-and-Ship strategy reduces onset-to-puncture time in more regions optimized with Drip-and-Ship. This reduction in onset-to-puncture time results in better patient outcomes and closes the gap between the Mothership and Drip-and-Ship strategies as patients can receive the benefits of early alteplase without having to delay the start of the EVT procedure. From a cost perspective, the addition of inter-facility air transfer in the Drip-and-Ship strategy rarely results in a more expensive alternative to the Mothership strategy. In fact, when the Drip-and-Ship strategy is modeled with a high probability of inter-facility transfer via air, it is often the least expensive transport strategy despite the high costs associated with air transportation. This is due to the nature of the Drip-and-Ship strategy as it allows for confirmation of the LVO diagnosis as well as EVT eligibility before further transport takes place. For these situations where the distance to the EVT-enabled facility is long, the consideration of air transport from scene of stroke can be a way to reduce the time to treatment and allow for better outcomes with the Mothership strategy; however, this will come with a greater cost of transport, as all patients suspected of an LVO in the field will be transported.

Efficiency at the alteplase-only facility remains a large factor in patient outcomes when choosing between the Mothership and Drip-and-Ship strategies. The benefit of inter-facility via air is realized more in a system that includes an efficient alteplase-only facility. This is especially evident in the results of the 240-min increment of the efficient Scenarios C and D, where a region outside the white arc is color coded red, indicating that Drip-and-Ship is preferred by both objectives. This result is surprising, as patients in this region are no longer eligible for alteplase treatment at the alteplase-only facility; however, their outcome is still optimized with the Drip-and-Ship Strategy as a result of efficiencies at the alteplase-only facility and a high probability of inter-facility transfer via air.

The symptom onset-to-first-medical-contact time is modeled as 20 min in all scenarios. However, this time varies significantly on a case-by-case basis. The general nature of the temporospatial diagrams allows for the model results to be extrapolated for patients with a prolonged onset-to-first-medical-contact time. Each concentric circle in the diagram represents 5 min of driving time in the original interpretation of the results, however these can also be used to elongate the onset-to-first-medical-contact time assumption.

This study only considers the cost of transportation from scene of stroke to the destination hospital and transfers between hospitals. The potential cost savings due to improved patient outcome was not incorporated into this model. This study has defined good outcomes as a 90-day mRS (modified Rankin score) of 0 or 1, and studies have shown that lower 90-day mRS results in lower health system costs (22). Therefore, for those areas where the transport strategy was divergent between patient outcomes and transport costs, these differences would likely not exist if full system costs were considered.

The comparison of drip-and-ship and mothership has been done using a randomized controlled trial design in Catalonia Spain with the RACECAT (Direct Transfer to Endovascular Center of Acute Stroke Patients with Suspected Large Vessel Occlusion in the Catalan Territory) Trial (23). The full results from the trial are still pending publication, but preliminary results have been presented and show neutral results (24). In RACECAT, the PSCs were generally close to the CSCs (within 1 h); however, the results from the trial are consistent with these modeled results, as it shows that patients in some regions benefit using drip-and-ship while other patients in other regions benefit with mothership. Furthermore, the best transport destination is also dependent on stroke system efficiency.

There are limitations to the model and data within it. We have assumed lead times and air ambulance logistics which correspond to rotary wing air transport, whereas fixed wing transport differs logistically from rotary wing but is used to transport some stroke patients between facilities for EVT treatment. Inter-facility transport is modeled with the assumption that when air transport is unavailable, ground transport is mobilized to transfer the patient, regardless of the driving distance. There may be cases where the ground driving distance is too long and the patient is not transported for EVT treatment if the air transport is not an option. Furthermore, we have assumed that the distance via ground transport is the same as air transport, which is not always the case, and ground transport distances are often larger than the air transport distance, which biases the results toward greater ground transport in areas closer to the endovascular-enabled center. We have also assumed equivalent door-in-door-out (DIDO) times for patients with known contraindications to alteplase (beyond the 4.5-h threshold) as those patients without. Patients with known contraindications are likely to move through the alteplase-only facility quicker than those without, which would impact their onset-to-puncture times. There is some emerging evidence that show that rural patients arrive at hospital later than urban patients (25). This may mean that for our scenarios the onset to first medical contact (time of ambulance arrival at scene of stroke) is longer for regions close to the PSC and shorter for regions closer to the CSC. Further addition to the model can be done in future work to account for this; however, it should be noted that this model's population are those patients that arrive by ambulance using an LVO screening tool. Typically, ambulance is used in early symptom recognition.

This model is based on the decay curve for thrombolysis with alteplase. There is emerging evidence that Tenecteplase is not inferior to alteplase in treating acute ischemic stroke patients (26, 27). If Tenecteplase is used in place of alteplase treatment, the results presented here may change depending on the association between time to treatment and outcomes with Tenecteplase. Furthermore, there are some potential process improvements that Tenecteplase may offer, since it is a single bolus delivery with no infusion.

## Conclusions

Both patient outcome and transport cost can largely be optimized with the same transport decision for suspected LVO ischemic stroke patients whose symptom onset occurs outside the direct catchment area of an EVT-enabled facility. A preference toward Drip-and-Ship occurs in more regions when efficiency at the alteplase-only facility is optimal and there is a high probability of inter-facility transfer via air. Divergent regions do exist where patient outcome and transport cost cannot be optimized with the same transport decision. However, in these regions, our analyses have shown marginal differences between in the divergence. Transport decisions in divergent regions should remain outcome focused as the marginal differences can likely be recovered as there are downstream hospital cost savings associated with better patient outcomes.

## Data Availability Statement

The raw data supporting the conclusions of this article will be made available by the authors, without undue reservation.

## Author Contributions

AW conceptualized the study, wrote the code, and wrote the first version of the manuscript. PF provided details about air transport in Canada, provided realistic scenarios for the study, and provided critical review of the manuscript. JH provided details to the patient outcomes model, the visualizations, and provided critical review of the manuscript. PV provided assistance with the methodology and provided critical review of the manuscript. DV provided details about the process involved for endovascular therapy and provided critical review of the manuscript. NK supervised the study, provided funding for the study, wrote parts of this manuscript, and provided feedback on the methods for this study. All authors contributed to the article and approved the submitted version.

## Funding

This study was funded by CIHR (Canadian Institutes of Health Research) Project Grant 169124, which is titled, Atlantic Canada Together Enhancing Acute Stroke Treatment (ACTEAST): Improving Access and Efficiency of Treatment.

## Conflict of Interest

NK and JH are co-founders and part equity owner of DESTINE Health Inc. The remaining authors declare that the research was conducted in the absence of any commercial or financial relationships that could be construed as a potential conflict of interest.

## Publisher's Note

All claims expressed in this article are solely those of the authors and do not necessarily represent those of their affiliated organizations, or those of the publisher, the editors and the reviewers. Any product that may be evaluated in this article, or claim that may be made by its manufacturer, is not guaranteed or endorsed by the publisher.

## References

[1] Gomez CR. Editorial: time is brain! J Stroke Cereb Dis. (1993) 3:1–2. 10.1016/S1052-3057(10)80125-9 26487071

[2] Saver JL. Time is brain - Quantified. Stroke. (2006) 37:263–6. 10.1161/01.STR.0000196957.55928.ab 16339467

[3] National Institute of Neurological Disorders and Stroke rt-PA Stroke Study Group. Tissue plasminogen activator for acute ischemic stroke. N Engl J Med. (1995) 333:1581–7. 10.1056/NEJM199512143332401 7477192

[4] Hacke W Donnan G Fieschi C Kaste M von Kummer R Broderick JP. Association of outcome with early stroke treatment: pooled analysis of ATLANTIS, ECASS, and NINDS rt-PA stroke trials. Lancet. (2004) 363:768–74. 10.1016/S0140-6736(04)15692-4 15016487

[5] Berkhemer OA Fransen PSS Beumer D Van Den Berg LA Lingsma HF Yoo AJ. A randomized trial of intraarterial treatment for acute ischemic stroke. N Engl J Med. (2015) 372:11–20. 10.1056/NEJMoa1411587 25517348

[6] Jovin TG Chamorro A Cobo E De Miquel MA Molina CA Rovira A. Thrombectomy within 8 hours after symptom onset in ischemic stroke. N Engl J Med. (2015) 372:2296–306. 10.1056/NEJMoa1503780 25882510

[7] Saver JL Goyal M Bonafe A Diener HC Levy EI Pereira VM. Stent-retriever thrombectomy after intravenous t-PA vs. t-PA alone in stroke. N Engl J Med. (2015) 372:2285–95. 10.1056/NEJMoa1415061 25882376

[8] Goyal M Demchuk AM Menon BK Eesa M Rempel JL Thornton J. Randomized assessment of rapid endovascular treatment of ischemic stroke. N Engl J Med. (2015) 372:1019–30. 10.1056/NEJMoa1414905 25671798

[9] Campbell BCV Mitchell PJ Kleinig TJ Dewey HM Churilov L Yassi N. Endovascular therapy for ischemic stroke with perfusion-imaging selection. N Engl J Med. (2015) 372:1009–18. 10.1056/NEJMoa1414792 25671797

[10] Milne MSW Holodinsky JK Hill MD Nygren A Qiu C Goyal M. Drip 'n ship versus mothership for endovascular treatment: modeling the best transportation options for optimal outcomes. Stroke. (2017) 48:791–4. 10.1161/STROKEAHA.116.015321 28100764

[11] Holodinsky JK Williamson TS Demchuk AM Zhao H Zhu L Francis MJ. Modeling stroke patient transport for all patients with suspected large-vessel occlusion. JAMA Neurol. (2018) 75:1477–86. 10.1001/jamaneurol.2018.2424 30193366 PMC6583214

[12] Schlemm L Endres M Scheitz JF Ernst M Nolte CH Schlemm E. Comparative evaluation of 10 prehospital triage strategy paradigms for patients with suspected acute ischemic stroke. J Am Heart Assoc. (2019) 8:e012665. 10.1161/JAHA.119.012665 31189395 PMC6645624

[13] Venema E Lingsma HF Chalos V Mulder MJHL Lahr MMH Van Der Lugt A. Personalized prehospital triage in acute ischemic stroke: a decision-analytic model. Stroke. (2019) 50:313–20. 10.1161/STROKEAHA.118.022562 30661502 PMC6358183

[14] Bogle BM Asimos AW Rosamond WD. Regional evaluation of the severity-based stroke triage algorithm for emergency medical services using discrete event simulation. Stroke. (2017) 48:2827–35. 10.1161/STROKEAHA.117.017905 28916666 PMC5639945

[15] Xu Y Parikh NS Jiao B Willey JZ Boehme AK Elkind MSV. Decision analysis model for prehospital triage of patients with acute stroke. Stroke. (2019) 50:970–7. 10.1161/STROKEAHA.118.023272 30908159 PMC6435279

[16] Holodinsky JK Williamson TS Kamal N Mayank D Hill MD Goyal M. Drip and ship versus direct to comprehensive stroke center. Stroke. (2017) 48:233–8. 10.1161/STROKEAHA.116.014306 27899757

[17] Whelan KR Hamilton J Peeling L Graham B Hunter G Kelly ME. Importance of developing stroke systems of care to improve access to endovascular therapies. World Neurosurg. (2016) 88:678–80. 10.1016/j.wneu.2016.02.098 26944889

[18] Holodinsky JK Kamal N Zerna C Ospel JM Zhu L Wilson AT Hill MD Goyal M. In what scenarios does a mobile stroke unit predict better patient outcomes? A modeling study. Stroke. (2020) 51:1805–12. 10.1161/STROKEAHA.119.028474 32389068

[19] Lawless J Tallon JM Petrie D. Aborted air medical missions: a 4-year quality review of a Canadian province-wide air medical program. Air Med J. (2005) 24:79–82. 10.1016/j.amj.2004.12.006 15741954

[20] Yan C Zheng Y Hill MD Mann B Jeerakathil T Kamal N. Health technology optimization analysis: conceptual approach and illustrative application. MDM Policy Pract. (2018) 3:238146831877480. 10.1177/2381468318774804 30288446 PMC6157433

[21] Achit H Soudant M Hosseini K Bannay A Epstein J Bracard S. Cost-effectiveness of thrombectomy in patients with acute ischemic stroke: the THRACE randomized controlled trial. Stroke. (2017) 48:2843–7. 10.1161/STROKEAHA.117.017856 28916667

[22] Kim SE Lee H Kim JY Lee KJ Kang J Kim BJ. Three-month modified Rankin Scale as a determinant of 5-year cumulative costs after ischemic stroke: an analysis of 11,136 patients in Korea. Neurology. (2020) 94:e978–91. 10.1212/WNL.0000000000011295 32029544

[23] Abilleira S Pérez de la Ossa N Jiménez X Cardona P Cocho D Purroy F. Transfer to the local stroke center versus direct transfer to endovascular center of acute stroke patients with suspected large vessel occlusion in the Catalan territory (RACECAT): study protocol of a cluster randomized within a cohort trial. Int J Stroke. (2019) 14:734–44. 10.1177/1747493019852176 31142219

[24] Desai SM Leslie-Mazwi TM Hirsch JA Jadhav AP. Optimal transfer paradigm for emergent large vessel occlusion strokes: recognition to recanalization in the RACECAT trial. J NeuroIntervention Surg. (2021) 13:97–9. 10.1136/neurintsurg-2020-017227 33500255

[25] Lim CD Ryoo HW Hwang YH Lee MJ Shin SJ Ahn JY. Urban-rural gap in the prehospital delay of acute stroke patients. J Korean Soc Emerg Med. (2013) 24:664–73. 10.1161/STROKEAHA.120.029318 32833593

[26] Campbell BC Mitchell PJ Churilov L Yassi N Kleinig TJ Dowling RJ. Tenecteplase versus alteplase before thrombectomy for ischemic stroke. N Engl J Med. (2018) 378:1573–82. 10.1056/NEJMoa1716405 29694815

[27] Logallo N Novotny V Assmus J Kvistad CE Alteheld L Rønning OM. Tenecteplase versus alteplase for management of acute ischaemic stroke (NOR-TEST): a phase 3, randomised, open-label, blinded endpoint trial. Lancet Neurol. (2017) 16:781–8. 10.1016/S1474-4422(17)30253-3 28780236

